# Primary Ureteral Hemangiosarcoma in a dog

**DOI:** 10.1186/s12917-020-02609-8

**Published:** 2020-10-12

**Authors:** Joseph A. Polit, Elisabeth V. Moore, Ember Epperson

**Affiliations:** 1 Chesapeake Veterinary Surgical Specialist, 1209 Cromwell Bridge Rd, Towson, Maryland 21286 USA; 2 Antech Diagnostics, 1111 Marcus Avenue, Lake success, New York, 11042 USA

**Keywords:** Ureteral neoplasm, Ureteral hemangiosarcoma, Hydroureter, Dog

## Abstract

**Background:**

Primary ureteral neoplasia in dogs is extremely rare. To the best of the authors’ knowledge, this is the second documented case of a primary ureteral hemangiosarcoma. This case report describes the clinical and pathological findings of a primary distal ureteral hemangiosarcoma.

**Case presentation:**

A 12-year-old spayed female goldendoodle was presented with a history of polyuria and weight loss. Abdominal radiographs revealed a large cranial abdominal mass. Abdominal ultrasound and computed tomography (CT) identified a left sided distal ureteral mass with secondary hydroureter and a left lateral hepatic mass with no evidence of connection or diffuse metastasis. A left ureteronephrectomy, partial cystectomy, and left lateral liver lobectomy were performed. Histopathology was consistent with primary ureteral hemangiosarcoma and a hepatocellular carcinoma. Adjunctive therapy including chemotherapy was discussed but declined.

**Conclusion:**

Due to its rarity, the authors of this case presentation believe that ureteral hemangiosarcoma should be included as a differential diagnosis when evaluating a ureteral mass. With the unknown, and suspected poor prognosis, routine monitoring with adjunctive therapy should be considered.

## Background

Primary ureteral neoplasia in dogs is extremely rare [1, 2]. This case report describes the clinical and pathological findings of a primary distal ureteral hemangiosarcoma. Previous malignant and benign cases of ureteral neoplasia have been reported, including transitional cell carcinoma, fibroepithelial polyps, leiomyoma, leiomyosarcoma, spindle cell sarcoma, and hemangiosarcoma [2–9]. To the best of the authors’ knowledge, this is the second documented case of a primary ureteral hemangiosarcoma [9].

## Case presentation

A 12-year-old, 23 kg, spayed female Golden Doodle; that was privately owned was presented for evaluation of a large cranial abdominal mass as well as a firm caudal abdominal mass. Historical clinical signs included polyuria and progressive weight loss for 1–2 months. Biochemistry performed through the primary care veterinarian revealed an elevated alkaline phosphatase (ALP) of 2181 U/L (reference range: 20 to 150 U/L), and an elevated alanine aminotransferase (ALT) of 303 U/L (reference range: 10 to 118 U/L). Radiographs and ultrasound indicated there was a large cranial abdominal mass suspected to be associated with the liver and a second caudal abdominal mass with an unknown origin that appeared to be compressing the urinary bladder.

On presentation, the dog was bright, alert, and responsive. Body condition score was 4/9. The patient was non-painful on abdominal palpation. Abdominal palpation revealed a cranial abdominal mass effect as well as a firm irregular caudal abdominal mass. The remainder of the physical examination was unremarkable. Hematology was unremarkable. The serum biochemistry revealed an elevated alkaline phosphatase (ALP) of 2102 U/L (reference range: 20 to 150 U/L), and an elevated alanine aminotransferase (ALT) of 299 U/L (reference range: 10 to 118 U/L). Urine analysis demonstrated 3 + protein, with a urine specific gravity of 1.050. A coagulation profile was performed, which was unremarkable. Three-view thoracic radiographs did not show any signs of metastasis or any other significant abnormalities.

**Fig. 1:**
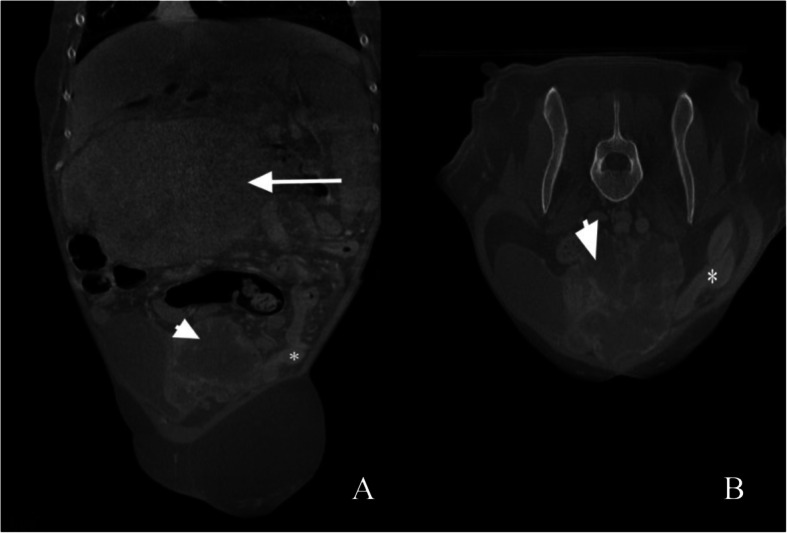
Dorsal (**a**) and transverse (**b**) reconstructed postcontrast, soft tissue algorithm, computed tomography images of the abdomen. Hydroureter (*) secondary to an expansile obstructive mass (short arrow) of the left ureter is visible as well as an attenuating mass of the left lateral liver lobe (long arrow)

**Fig. 2:**
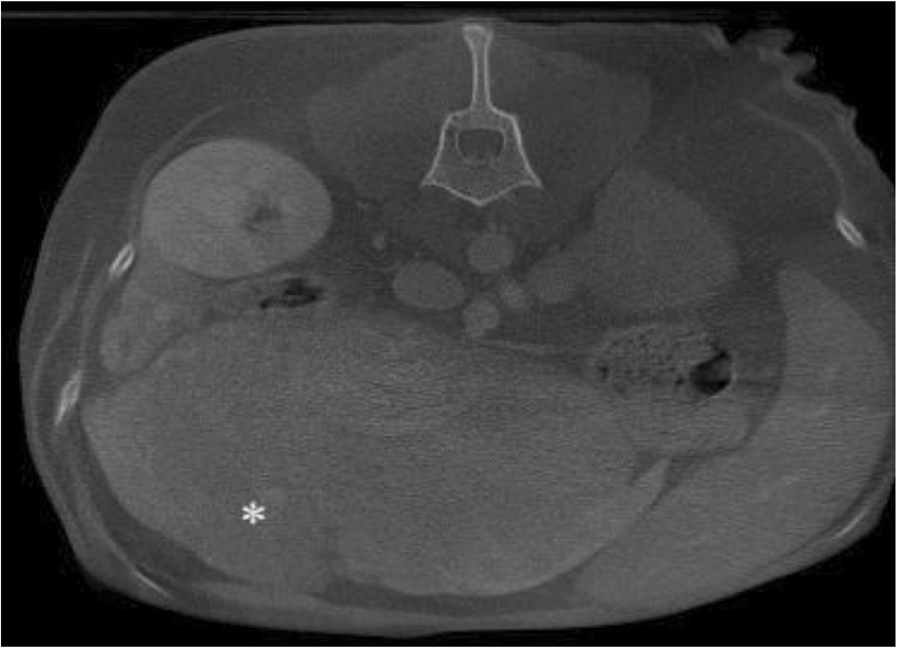
Transverse reconstructed postcontrast, soft tissue algorithm, computed tomography image of the abdomen. Attenuating mass of the left lateral liver lobe (*)

An abdominal computed tomography (CT) scan showed a well-defined, irregularly marginated, multilobulated, hypoattenuating mass, measuring 4.7 cm x 6.8 cm x 8 cm, at the level of the left distal ureter and ureteral papilla, with secondary severe left proximal ureteral dilation, measuring 0.5–0.6 cm (Fig. 1). The cranial abdomen revealed a well marginated, mildly heterogenous, contrasting soft tissue attenuating mass, measuring 6.3 cm x 12.5 cm x 10.9 cm, arising from the caudal aspect of the left lateral liver lobe (Fig. 2).

**Fig. 3:**
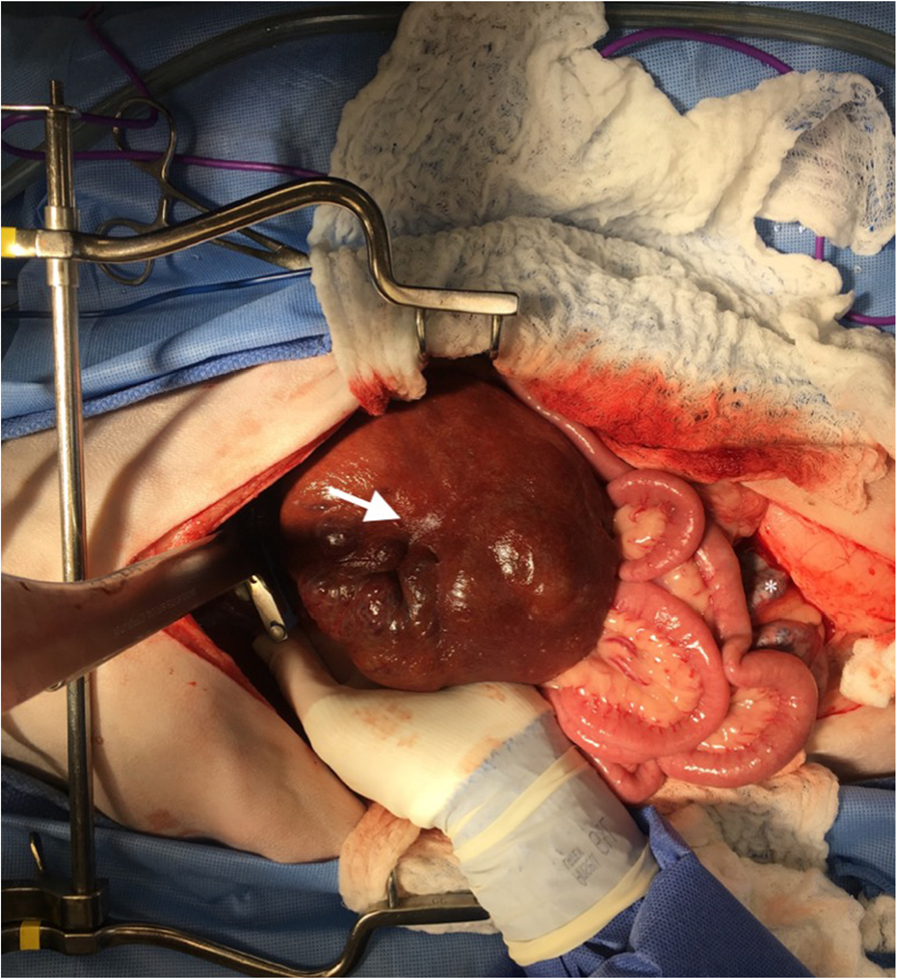
Photograph of left lateral liver mass prior to excision

**Fig. 4:**
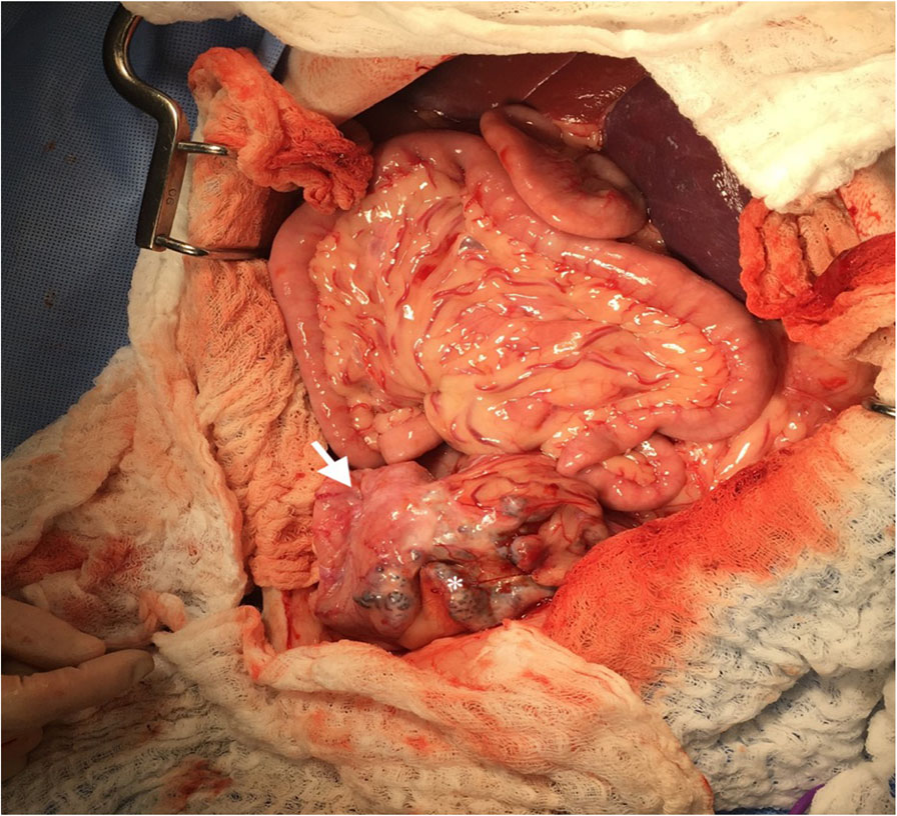
Photograph of left ureteral mass (*) prior to excision

An exploratory celiotomy was performed. The celiotomy identified a large mottled mass, measuring 18 cm x 10 cm x 4 cm associated with the left lateral liver lobe (Fig. 3), along with a firm, nodular, multilobular mass, measuring 8 cm x 5 cm, associated with the distal left ureter attached to the left aspect of the urinary bladder (Fig. 4) with proximal ureteral dilation. A left lateral liver lobectomy was performed with a TA-55 stapler and the Ligasure Precise vessel sealing device. The left ureter mass was dissected out and found to be adhered to the bladder serosa and found to enter the bladder lumen through the left ureteral papilla. A left sided ureteronephrectomy and partial cystectomy were performed to remove the mass en bloc. No other abnormalities were noted on final exploration. The patient underwent a successful and uneventful recovery and was discharged from the hospital three days postoperatively. Adjunctive therapy including chemotherapy was discussed but declined. At the initial postoperative evaluation (12 days), the owner reported no overt issues and improvement in appetite and energy, with no abnormalities noted on physical exam. At subsequent evaluations (30-days, 40-days and 120-days), the patient is reported to show consistent and progressive status improvement with no concerns.

**Fig. 5:**
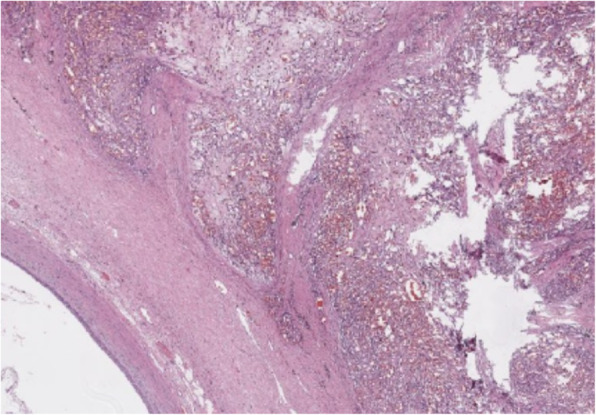
Vascular neoplasm within the wall of the ureter, with markedly dilated ureteral lumen at bottom left

**Fig. 6:**
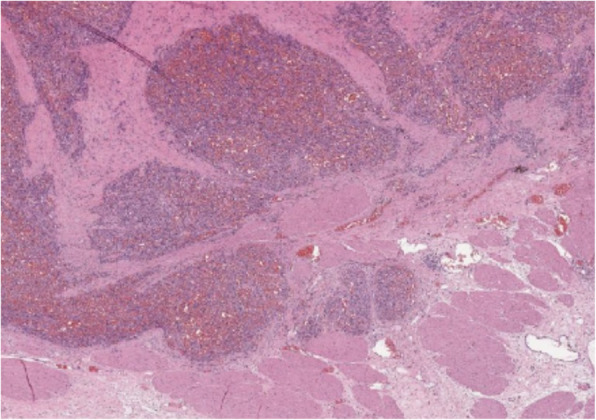
Poorly demarcated mass invading prominent smooth muscle of the ureteral papilla

**Fig. 7:**
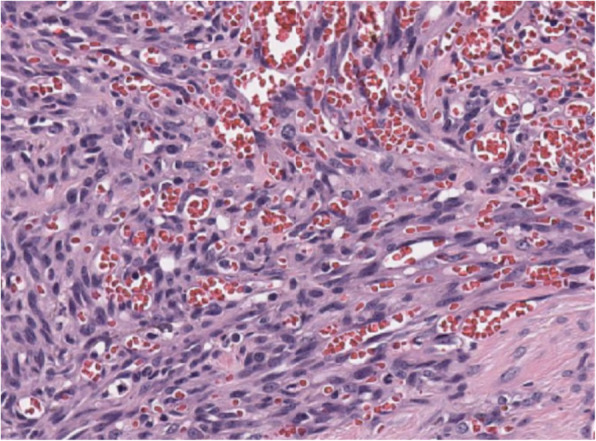
Neoplastic spindle cells form irregular blood-filled vascular channels

**Fig. 8:**
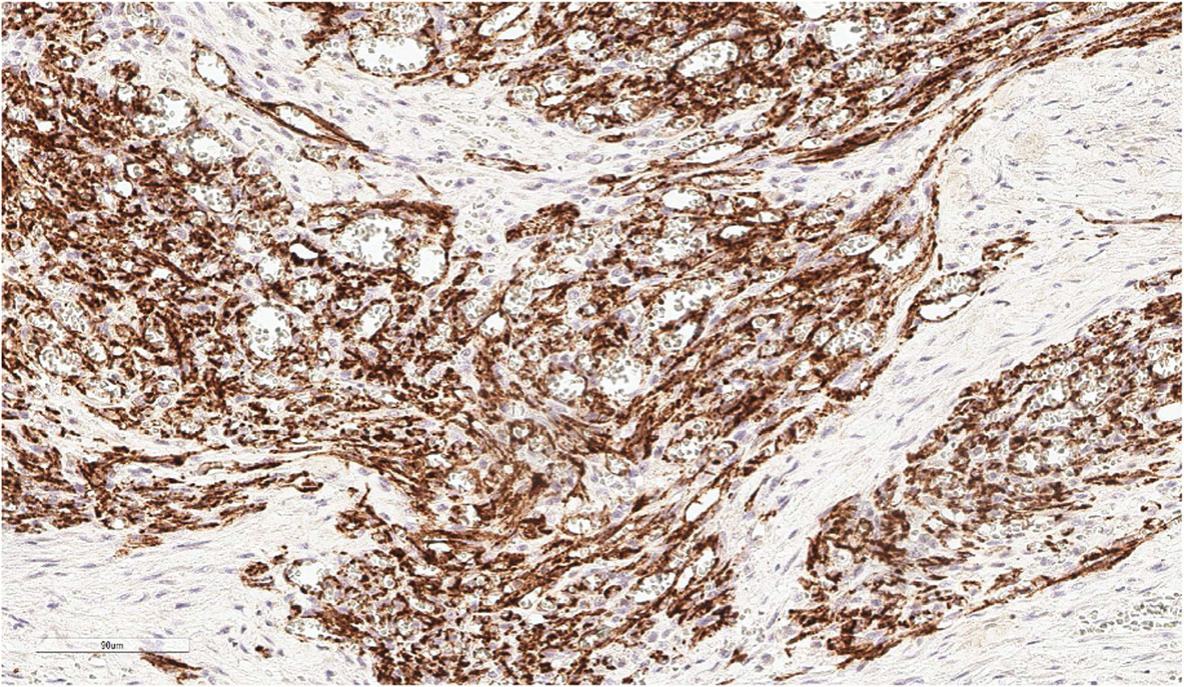
Neoplastic spindle cells exhibiting cytoplasmic and membranous staining for CD31 antigen

Histopathology was performed by a board-certified pathologist and confirmed by a second pathologist. The ureteral mass was narrowly excised with 0.1 mm surgical margins. Histologic findings of the ureteral mass (Fig. 5) revealed a densely cellular, poorly demarcated (Fig. 6), unencapsulated, infiltrative, variably cystic, and hemorrhagic mass which variably borders foci of marked hemorrhage, fibrin, edema, and necrosis. The cells are plump and spindloid, with indistinct cellular borders and wispy eosinophilic cytoplasm (Fig. 7). The nuclei are oval to elongate, with finely stippled to vesicular chromatin and one to three nucleoli. Anisocytosis and anisokaryosis are moderate, with a mitotic count of 6 in ten 400x fields. Immunohistochemistry was then performed revealing positive CD31 staining of the neoplastic spindle cells confirming ureteral hemangiosarcoma (Fig. 8).

**Fig. 9:**
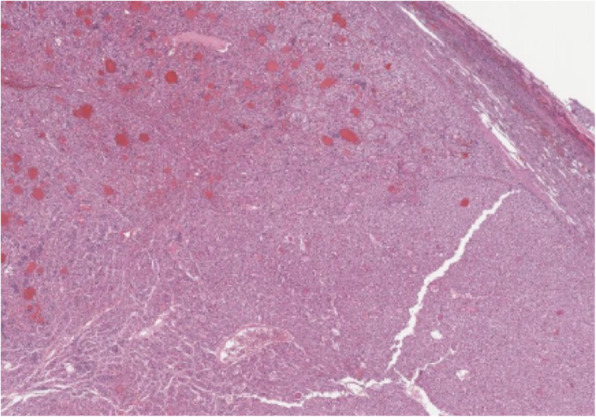
A densely cellular infiltrative mass locally replaces the hepatic parenchyma

**Fig. 10:**
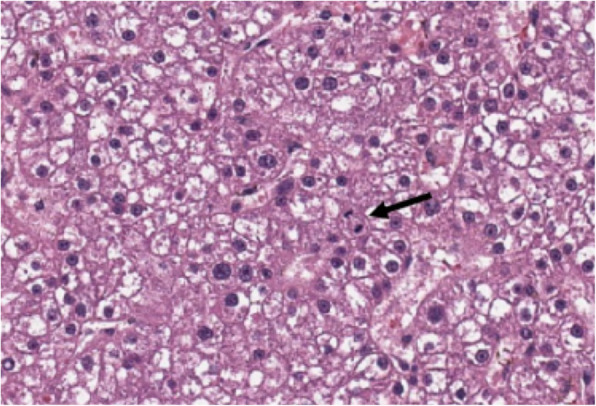
Foamy vacuolated polygonal cells form irregular broad trabeculae and cords (note mitotic figure at arrow)

Histologic findings of the liver mass revealed a densely cellular, poorly demarcated, unencapsulated, infiltrative, variably hemorrhagic, and necrotic mass (Fig. 9). The cells form coalescing broad hepatic cords and trabeculae, supported by a small amount of fibrovascular stroma (Fig. 10). Portal areas and central veins are not discernible within the mass. The cells are polygonal, with distinct cellular borders and a moderate to abundant amount of granular eosinophilic cytoplasm, which variably contains ill-defined vacuolar regions of clearing. The nuclei are round to oval with finely stippled chromatin and one to four nucleoli. Anisocytosis and anisokaryosis are moderate, and 11 mitotic figures are observed in ten 400x fields. The findings observed were confirmed to be consistent with hepatocellular carcinoma.

## Discussion and conclusion

Ureteral neoplasia is rare in dogs with the majority of tumors being benign [1, 10]. There is only one other recent documented case of ureteral hemangiosarcoma located in the proximal ureter [9]. Previously reported ureteral tumors include leiomyosarcoma, fibroepithelial polyps, fibropapilloma, transitional cell carcinoma, mast cell tumors, giant cell sarcoma and leiomyoma [2, 4, 6, 8, 11–15]. Hemangiosarcoma is a well-known malignant tumor originating from vascular endothelial cells [2, 9, 16, 17]. The most common primary sites reported for hemangiosarcoma include the spleen, right atrium, liver, and skin (particularly the dermis and subcutis) [9, 17, 18]. The canine urinary tract being a primary site of hemangiosarcoma is extremely rare [9, 19, 20]. Previous reports of hemangiosarcoma originating from the urinary tract were localized to the retroperitoneal space, kidney, urethra, prostate, and proximal ureter [9, 21–25].

In this case report, abdominal CT confirmed the presence of a distal ureteral mass with secondary hydroureter. As in people, distal ureteral masses in dogs are more likely to be malignant, whereas proximal ureteral masses are more likely to benign [4, 9, 12, 26, 27]. The histopathology results in this case report of malignant tumor of the distal ureter are supported by these previous findings.

Clinical signs of a ureteral mass may include polyuria, polydipsia, hematuria, urinary incontinence, abdominal pain, lethargy, weight loss, and anorexia [6, 9–11]. Clinical signs present with this case included only weight loss and polyuria. Unlike the previous case report of ureteral hemangiosarcoma [9], our patient was not noted to have hematuria. The vague clinical signs associated with a ureteral mass are broad and may be attributed to a multitude of different diseases, warranting a complete and thorough diagnostic evaluation as necessary for an accurate diagnosis [9].

Similar to the previously documented case of ureteral hemangiosarcoma, our patient’s pre-operative thoracic radiographs did not reveal any signs of metastatic disease indicating our patient was a suitable candidate for an exploratory celiotomy [9]. Treatment options for a ureteral mass commonly include surgical resection, specifically by ureteroneocystostomy or ureteronephrectomy [6, 9, 28, 29].

The literature reveals a range in prognosis associated with malignant ureteral masses, which are dependent upon the type of cancer [3, 7, 10, 14]. In the previously documented case of ureteral hemangiosarcoma, the patient was euthanized 40 days post-operatively due to dyspnea secondary to pulmonary metastasis [9]. In the presented case, the 40-day postoperative evaluation revealed consistent and progressive improvement in status, with no overt issues noted on the owners report. Unfortunately, due to the lacking existing evidence of prognosis and prognostic factors attributable to ureteral hemangiosarcoma, authors cannot predict the outcome of our patient, nor provide any extrapolations for future cases.

This case marks only the second documented case of ureteral hemangiosarcoma in canines. Due to its rarity, various clinical signs, and suspected poor prognosis, the authors of this case presentation believe that ureteral hemangiosarcoma should be included as a differential diagnosis when evaluating all ureteral masses despite the presence of hematuria. Currently, ureteral hemangiosarcoma has an unknown and suspected poor long term prognosis [9]. Routine monitoring with the addition of chemotherapy should be considered.

## Data Availability

Not applicable.
